# Practical Observations on the Use of Cold in the Treatment of Disease

**Published:** 1836-10

**Authors:** 


ART. XIII. *Erfahrungen ueber die Anwendung der Kälte in Krankheiten.* Von J. D. BRANDIS, M. D. Königl. Dänischen Leibarzte, &c. &c. &c. S. 116. *Berlin*, 1833.

*Practical Observations on the Use of Cold in the Treatment of Disease.* By J. D. BRANDIS, M. D. Physician in Ordinary to the King of Denmark, &c. &c. Pp. 116. *Berlin*, 1833.

THE title of this work gives a very false idea of the nature of its contents: it has but little of a practical character about it; and even that little is so obscured and contaminated, (if it be allowed to use the expression,) by peculiar dogmatic views in physiology and pathology, that the strongest impression made on the mind of the reader must be regret that the fifty years' attention which the author assures us he has devoted to this particular subject, should not have been guided by philosophical principles, of less pretension perhaps, but at the same time resting on clearer evidence and of more extended applicability.

A brief notice of it in the pages of this Journal may, notwithstanding, offer some interest, were it only as illustrating by a specimen the mode of reasoning and the nature of the doctrines which prevail among a certain class of German transcendentalists, on the obscure and difficult matters occupying a position intermediate between metaphysics and the natural sciences.

As is very commonly the case with those who assume the greatest latitude in their hypothetical speculations, and in the extent of the inferences deduced from them, Dr. Brandis discourses most rationally at the outset on the futility, for all truly philosophical purposes, of the attempt to separate theory and experience,—and on the importance of the accessory sciences of Anatomy, Physics, Chemistry, &c. He soon, however, quits this moderate tone, and, commencing by an eulogium on Hippocrates, not merely as a model of unrivalled excellence in observation and natural sagacity, but as a guide still meriting implicit confidence in the modes of philosophical investigation, proceeds thus:

"He, (Hippocrates,) it is true, was acquainted neither with galvanism nor magnetism, with oxygen nor nitrogen; but, not the less, had a clear perception of the *pneuma* which produces the unity and mutual dependence of the universe and the individual organism,—and of its influence on climate, health, and disease. He ventured to assert that anatomy was a study rather for painters than physicians; and yet was tolerably familiar with the functions of organs and their mutual re-actions on each other. His Calidum and Frigidum, Humidum and Siccum, had no foundation in an accurate knowledge of vital action,—and yet it is his observation, (true to nature,) of increase and diminution of animal and vegetative life, that, subsequently, and in forms against which he protested, has been reproduced by others, and made the basis of theories of every kind." *Introd.* p. v.

Following the example he has thus proposed to himself, the author next announces, as the leading theory of his treatise, the following proposition:

"That life itself produces heat and cold in the organism, in the same manner that the cosmic life (*pneuma*,) causes both in the universe, i. e. directly, and not as a consequence of the combinations or decompositions of matter." P. vii.

This is but a small portion of the powers attributed to this vital principle; for, by implication at least, we find bestowed upon it, intelligence; discrimination; volition; the regulation of all organic processes with the purpose of preserving the integrity of the œconomy;—a capability of counteracting the deteriorating influence of external agents; and, at the same time, a susceptibility of certain changes in the nature and direction of its activity from the operation of various causes.

To attempt to follow out in their full extent the conclusions which Dr. Brandis goes on to deduce from these and similar principles, would be a very unprofitable task, and, in truth, would be attended with no small difficulty, owing to the impossibility of conveying in a moderate compass any correct notion of his opinions, even when they admit of comprehension. It will be enough to give a sketch of the manner in which he applies them to the subject under consideration. Assuming that the causes of disease operate directly upon the vital principle, or, as he terms it, the organic will, he asserts that diseases admit of cure by giving another direction to this will, through the medium of influences to which he attaches the common appellation of counter-irritants. Of these the most speedy and effective, whether as regards the entire organism, or individual parts of it, consists in change of temperature. Cold, in particular, excites the organic will to the production of heat, and thereby moderates or removes morbid vital manifestations. Again, the absolute effects of cold are of two kinds;—direct upon the particular parts to which it is applied; and indirect, by means of sympathies, on remote organs. In general terms, it first diminishes vital action, re-action succeeding, unless the application be so long continued as to exhaust and overcome the organic powers, and produce muscular in-action, stupor, sleep, &c. The extent of the effect produced admits of considerable variation, according to the rapidity of the transition from one vital condition to another, both as regards the direct and indirect consequences of the agent.

As relates to the vital manifestations which occur in certain organs vicariously with the applications of cold to others, they are more decided in proportion as the ordinary condition of the individual constitution is more excitable and indeterminate, as is the case in hot climates, in children, and during periods of change, *e. g.* dentition, approach of puberty, pregnancy, &c. Here also, as in the former case, much depends on the rapidity and the amount of the change of temperature, with reference to time as well as to degrees of heat. Pp. 21, 30.

Such is a brief abstract of the therapeutical doctrines of Dr. Brandis, as regards the effect of cold, stripped of the peculiar language and manner of expression by which his treatise is distinguished. It will be seen that, so far as they extend, they are more rational than might have been anticipated from some of his philosophical and pathological notions. They are confined, however, and scarcely reach beyond the bounds of the most ordinary common-place. At least, two points are passed over, which, in an attempt to estimate the powers and the mode of action of cold, as a therapeutic mean, would certainly deserve especial attention, viz. the internal use of cold, and the effects produced immediately upon the nervous system, in consequence of the sensations connected with its employment.

In typhoid fevers, Dr. Brandis has employed, since 1784, cold affusion and bathing with cold water, either alone or mixed with vinegar; and with greater success in proportion to the intensity of the heat of the skin, especially if accompanied with a small, hard pulse; whilst, on the contrary, he has found them less serviceable, or even injurious, in bilious and other fevers, in which the activity of the disease is concentrated on the intestinal canal. His experience on this practice was published as long back as 1794, in Hecker's Magazine, vol. v., and, as he states, previous to obtaining any intimation of the observations of Wright and Currie.

The notice of the application of cold in thoracic diseases is brief, and bears little trace of experience. It is needless to say that, in this country, the practice of using cold externally has been confidently recommended in certain pulmonary affections, chiefly of a chronic nature. Generally, the intention, as far as may be judged from the empirical nature of the practice, appears to be either to produce a certain degree of counter-irritation,—stimulants, as vinegar, &c. being applied in conjunction with cold water; or, by lessening the susceptibility of the external surface, to produce a tonic effect, and to lessen the liability to internal congestion from casual variations of external temperature. A third method, differing from either, consists in the continued application of cold in various shapes, with the view of producing sedative effects on the thoracic circulation, by the direct abstraction of heat: a mode of practice that finds a parallel in the employment of cold applications in peritoneal inflammations, as advocated by Dr. Sutton. This plan, though doubtless occasionally applicable, obviously requires caution, inasmuch as it is not without some risk of increasing internal disease, by repelling blood from the surface. The form of disease to which it appears best adapted, is acute pulmonary hemorrhage, accompanied by a hot skin, with a sense of internal heat, and an excited, but not forcible condition of circulation.

There appears to be something of novelty, at least, in the recommendation of cold bathing and affusions in rheumatism, a practice, of which Dr. Brandis speaks confidently, in very favorable terms, particularly with the intention of diminishing the susceptibility in certain individuals to changes of external temperature.

